# Cu-doped Nd_0.6_Sr_0.4_Co_1−*x*_Cu_*x*_O_3−*δ*_ (*x* = 0, 0.05, 0.1, 0.15, 0.2) as the cathode for intermediate-temperature solid oxide fuel cells

**DOI:** 10.1039/d3ra01469j

**Published:** 2023-05-23

**Authors:** Xu Du, Songbo Li, Shengli An, Liangmei Xue, Yang Ni

**Affiliations:** a School of Chemistry and Chemical Engineering, Inner Mongolia University of Science and Technology Baotou 014000 China songboli2021@hotmail.com; b School of Material and Metallurgical Engineering, Inner Mongolia University of Science and Technology Baotou 014010 China

## Abstract

Nd_0.6_Sr_0.4_Co_1−*x*_Cu_*x*_O_3−*δ*_ (*x* = 0, 0.05, 0.1, 0.15, 0.2) (NSCC_*x*_) was prepared by replacing Co with Cu. Its chemical compatibility, electrical conductivity, and electrochemical properties were studied by X-ray powder diffractometry, scanning electron microscopy, and X-ray photoelectron spectroscopy. The conductivity, AC impedance spectra, and output power of the single cell were tested in an electrochemical workstation. Results showed that the thermal expansion coefficient (TEC) and electrical conductivity of the sample decreased with the increase in Cu content. The TEC of NSCC_0.1_ decreased by 16.28% in the temperature range of 35 °C–800 °C, and its conductivity was 541 S cm^−1^ at 800 °C. Furthermore, a single cell was constructed with NSCC_*x*_ as the cathode, NiO-GDC as the anode, and GDC as the electrolyte. The peak power of the cell at 800 °C was 444.87 mW·cm^−2^, which was similar to that of the undoped sample. Compared with the undoped NSCC, NSCC_0.1_ showed lower TEC while maintaining its output power. Therefore, this material can be used as a cathode for solid oxide fuel cells.

## Abstract 2

Nd_0.6_Sr_0.4_Co_1−*x*_Cu_*x*_O_3−*δ*_ (*x* = 0, 0.05, 0.1, 0.15, 0.2) (NSCC_*x*_) was prepared by replacing Co with Cu.

## Graphical abstract

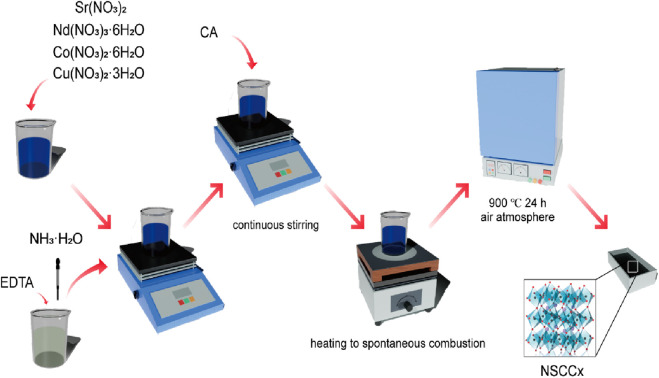

## 1. Introduction

A solid oxide fuel cell (SOFC) is a power generation device that directly converts chemical energy in fuel and oxidizer into electrical energy.^1,2^ SOFCs are energy efficient compared with other fuel cells and can run on a variety of fuels, making them an excellent candidate for a range of applications. However, SOFCs have not been widely popularized due to their high operating temperature, high cost, and short lifetime.^3,4^ Their high operating temperature is because SOFCs must be warmed up before use. In addition, the operating temperature above 1000 °C limits the materials available for this technology. Therefore, research on SOFCs focuses on reducing the operating temperature to expand the range of material selection and prolong the service life of full fuel cells.^5^ The polarization resistance of the electrode contributes 70% to 85% of the internal resistance of the full fuel cell. In addition, oxygen reduction is more difficult and requires higher activation energy than fuel oxidation at medium and low temperatures.^6^ Given that the polarization resistance of the cathode is greater than that of the anode, the performance of the former largely determines the performance of the whole SOFC.^7^ Therefore, a cathode material that can work at low temperature and provide excellent electrochemical performance must be developed.

Perovskite oxides with mixed ionic–electronic conductor properties are widely used for the cathodes of medium/low temperature SOFCs.^8,9^ This is particularly evident in perovskite oxides containing Co elements. La_1−*x*_Sr_*x*_CoO_3−*δ*_, SrCoO_3−*δ*_, BaCoO_3−*δ*_, and other perovskite oxides have high oxygen reduction reaction (ORR) activity and high electrical conductivity at low and medium temperatures, but their high thermal expansion coefficient (TEC) limits their application.^10,11^ The high TEC will cause the cathode to fall off during the full fuel cell operation, resulting in its failure. Replacing La^3+^ with Nd^3+^/Sm^3+^ that has a large radius can reduce the TEC while maintaining good electrochemical performance.^12^ Garibay *et al.*^13^ compared Nd_0.6_Sr_0.4_CoO_3−*δ*_ with La_0.6_Sr_0.4_CoO_3−*δ*_ and found that the former had lower TEC and conductivity but the same power density as the latter. Lee *et al.*^14^ found that the conductivity and TEC decreased successively from La to Gd in Ln_0.6_Sr_0.4_CoO_3−*δ*_ (Ln = La, Pr, Nd, Sm, Gd). Tamimi *et al.*^15^ found that materials containing Pr and Nd showed higher oxygen exchange rate and lower electrode impedance than those containing La in Ln_0.5_Sr_0.5_Co_0.8_Fe_0.2_O_3−*δ*_ (Ln = La, Pr, Nd), and this finding was directly related to the high oxygen mobility of the sample. Therefore, a compromise between electrocatalytic activity and TEC can be achieved by preparing a cathode containing the rare earth ion Nd.

In all kinds of perovskite oxides, the ionic radius and chemical state of B-site elements determine the ORR activity and electrocatalytic performance. Song *et al.*^16^ prepared Nd_0.5_Sr_0.5_Co_0.5_Mn_0.5_O_3−*δ*_ by doping Mn into Co and found that the use of an appropriate amount of Mn remarkably improved the performance of the material and produced the maximum power density of 592.80 mW cm^−2^ at 650 °C for a single cell. Yao *et al.*^17^ found that doping an appropriate amount of Cu in SrFe_0.9−*x*_Cu_*x*_W_0.1_O_3−*δ*_ (*x* = 0, 0.1, 0.2, 0.3) can improve the specific resistance of the material. These studies showed that the introduction of appropriate Cu can improve the electrochemical performance of the material and make it an ideal perovskite cathode for SOFCs.^18,19^

Till now, the electrochemical properties of Nd_0.6_Sr_0.4_Co_1−*x*_Cu_*x*_O_3−*δ*_ (*x* = 0, 0.05, 0.1, 0.15, 0.2) have not been reported. In this work, we prepared NSCC cathode with reduced TEC by maintaining the output power.

## 2. Experimental

### 2.1 Preparation

**Fig. 1:**
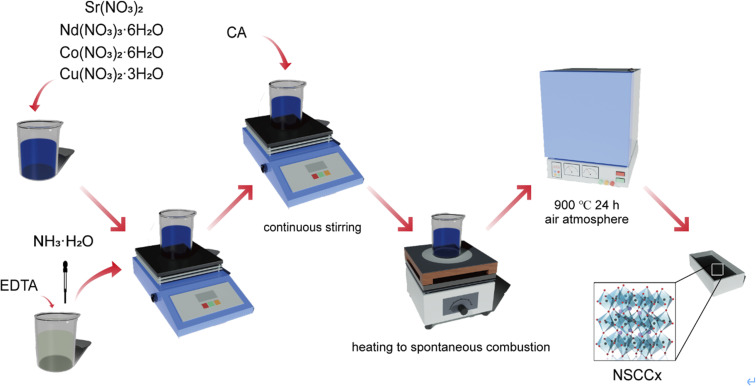
Preparation flow chart of NSCC_*x*_ samples.

Nd_0.6_Sr_0.4_Co_1−*x*_Cu_*x*_O_3−*δ*_ (*x* = 0, 0.05, 0.1, 0.15, 0.2) was prepared by EDTA–citric acid method as shown in Fig. 1. Nd(NO_3_)_3_·6H_2_O (Aldrich, 99%), Sr(NO_3_)_2_ (Aldrich, 99.99%), Co(NO_3_)_2_·6H_2_O (Aldrich, 99.99%), and Cu(NO_3_)_2_·3H_2_O (Aldrich, 99%) were added to deionized water and stirred until complete dissolution. EDTA was added to the nitrate solution and stirred for 1 h, followed by the addition of citric acid with stirring for 2 h (total metal ions : EDTA : molar ratio of citric acid = 1 : 1 : 2). The nitrate solution was adjusted to pH ≥ 6 by ammonia and stirred continuously at 80 °C until gel was formed. NSCC_*x*_ series cathode powders were prepared by heating the gel to spontaneous combustion and then heating the precursor again at 900 °C for 24 h in air atmosphere. NSCC_*x*_ (*x* = 0, 0.05, 0.1, 0.15, 0.2) was named as NSCC, NSCC_0.05_, NSCC_0.1_, NSCC_0.15_, and NSCC_0.2_ according to the Cu content.

Gd_0.20_Ce_0.80_O_1.90_ (GDC) was prepared by citric acid–nitrate method. The molar ratio of total metal ions to citric acid was 1 : 2. Gd(NO_3_)_3_·6H_2_O and Ce(NO_3_)_3_·6H_2_O were dissolved in deionized water, and citric acid was then added to the solution with stirring for 2 h at 80 °C until gel was formed. GDC powder was obtained after the gel was heated to spontaneous combustion and kept at 1450 °C for 10 h in an air atmosphere. For the evaluation of the thermal compatibility of NSCC_*x*_ and GDC, NSCC_*x*_ + GDC powder was prepared by ball milling with anhydrous ethanol for 15 h and calcinating at 900 °C for 5 h.

Electrochemical impedance spectroscopy (EIS) was performed in symmetrical cells supported by electrolytes. The GDC powder was pressed into sheets (diameter: 15 mm, thickness: 0.5 mm) at 200 Mpa and calcined at 1450 °C for 10 h to prepare the GDC electrolyte carrier. The cathode was synthesized by screen print. NSCC_*x*_ powder was mixed with terpinol (Aldrich, 95%) and ethyl cellulose (Aldrich, 45–55 Mpa s) at a mass ratio of 2 : 2 : 1 to prepare the cathode slurry. The cathode was printed symmetrically and then heated at 900 °C in air atmosphere for 5 h. Finally, a symmetry cell with the structure of NSCC_*x*_|GDC|NSCC_*x*_ was obtained. A single fuel cell with anode support structure was prepared by co-pressure method. GDC (0.15 g) was laid flat on the NiO-GDC (1.5 g), and then a NiO-GDC|GDC anode support half cell was obtained through the compaction of a 200 Mpa pressure. The preparation method of cathode side was the same as that of symmetrical fuel cell cathode, and the two electrodes were coated with silver slurry as the current collector for the next fuel cell performance test.

### 2.2 Characterization

The crystal structures of the samples were characterized by X-ray powder diffraction (XRD) (Netherlands, Malvern Panalytical, Empyrean) under radiation of Cu–Kα (*λ* = 1.5418 Å), current of 40 mA, voltage of 45 kV, and range from 20° to 90°. The morphologies were investigated by scanning electron microscopy (SEM) (Czech, TESCAN, GAIA3). The TEC was measured by L75HS 1600 (NETZSCH) thermal dilatometer at a temperature range of 30 °C–850 °C and a heating rate of 5 °C min^−1^. The weight loss of the material at 30 °C–850 °C was investigated by using TG-DTA (TG/DTA7300 Hitachi). X-ray photoelectron spectroscopy (XPS) was also conducted (Thermo Scientific Escalab 250Xi).

The electrochemical performance of NSCC_*x*_ was tested in an electrochemical workstation (PGSTAT302N Metrohm). Ceramic strip samples for TEC and electronic conductivity measurements were prepared by sintering NSCC_*x*_ samples in an air atmosphere at 900 °C for 5 h. The conductivity of the material was tested at 200 °C–800 °C by a DC four-electrode method, and the current was collected by silver slurry (DAD-87, Shanghai Research Institute of Synthetic Matrix) and silver wire (99.99%). Apply silver paste to the connecting part of the silver wire and the cathode bar to ensure good contact between the silver wire and the cathode strip. The electrochemical impedance of the material was measured at 650 °C–800 °C by symmetrical cell method at a frequency of 100 kHz–0.1 Hz, an amplitude of 10 mV, and RMS mode. Power density test was performed by using the single cell in the same temperature range as EIS with humidified hydrogen (3% H_2_O) as the anode fuel and air as the oxidizer.

## 3. Results and discussion

**Fig. 2:**
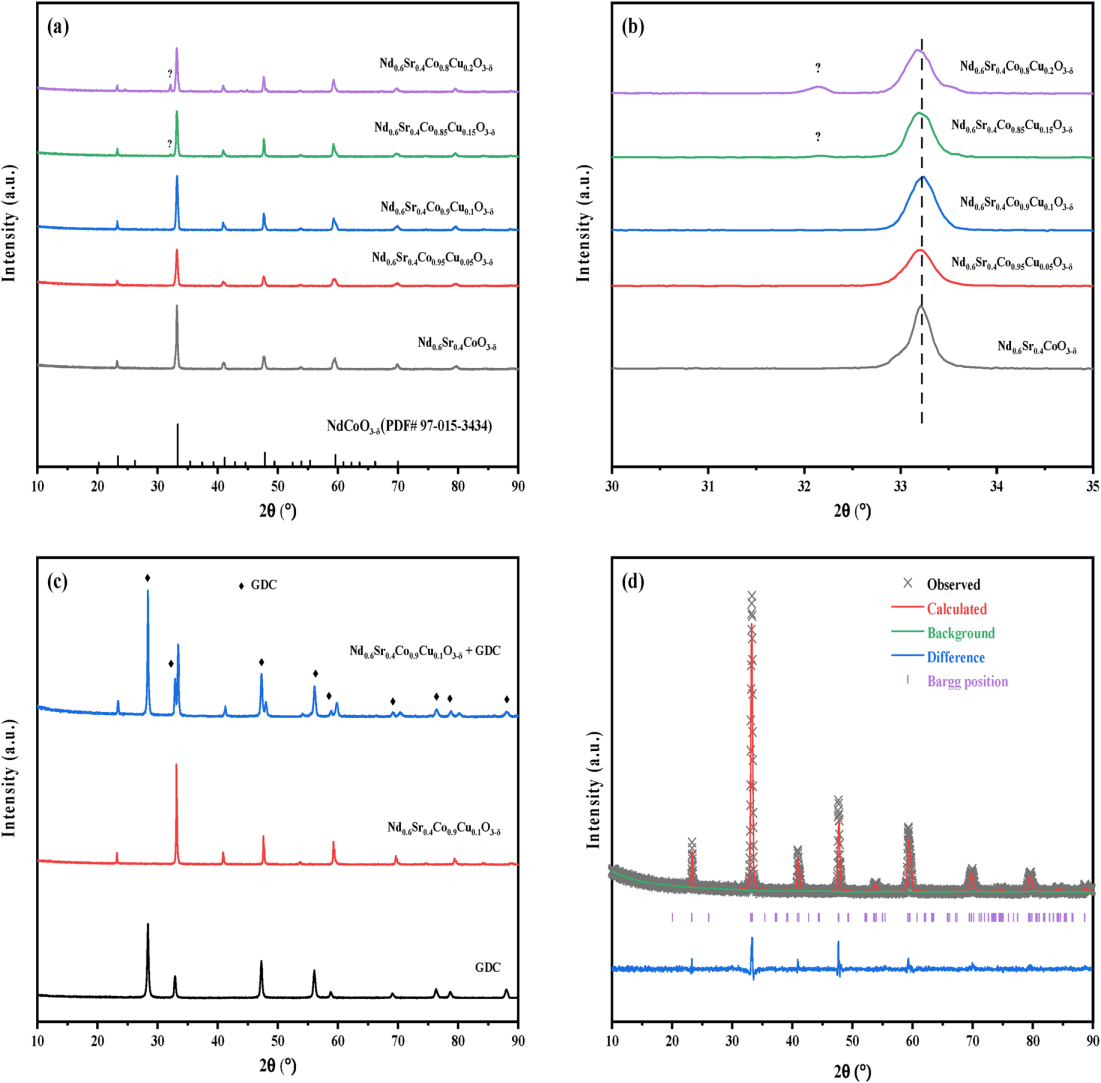
(a) XRD patterns of NSCC_*x*_ (b) enlarged patterns at 30–35°; (c) XRD patterns of GDC, NSCC_0.1_ and NSCC_0.1_ + GDC; (d) Rietveld patterns of NSCC_0.1_.

The XRD patterns of NSCC_*x*_ calcined at 900 °C for 24 h are shown in Fig. 2(a). The diffraction peaks of all samples have similar intensities and are consistent with the structure of NdCoO_3_ (PDF# 97-015-3434). An unknown diffraction peak is observed when the Cu content is greater than 0.15 as shown in Fig. 2(b). Owing to the existence of Cu and Co elements in many states, part of the valences and the ion radii of the spin state are close, and the principal diffraction peaks do not exhibit any deviation. GDC electrolyte and NSCC_0.1_ were mixed with equal mass ratio and calcined, and the XRD patterns are shown in Fig. 2(c). NSCC_0.1_ has no solid phase reaction with GDC, indicating their good chemical compatibility.^20^ The XRD patterns were further analyzed by Rietveld method according to *Pbnm* space group. The Rietveld refined map of NSCC_0.1_ is shown in Fig. 2(d). With the increase in Cu content, the crystal cell volume gradually increases as shown in Table 1.

**Table 1:** The Rietveld XRD results of NSCC_*x*_

Sample	Space group	Volume (Å)	*a* (Å)	*b* (Å)	*c* (Å)	*χ* ^2^	*R*_wp_ (%)	*R*_p_ (%)
NSCC	*Pbnm*	217.68	5.3417	5.3890	7.3562	1.355	5.96	4.72
NSCC_0.05_	*Pbnm*	219.08	5.3959	5.3480	7.5917	1.580	7.37	5.82
NSCC_0.1_	*Pbnm*	220.30	5.4055	5.3542	7.6120	1.747	7.18	5.58

**Fig. 3:**
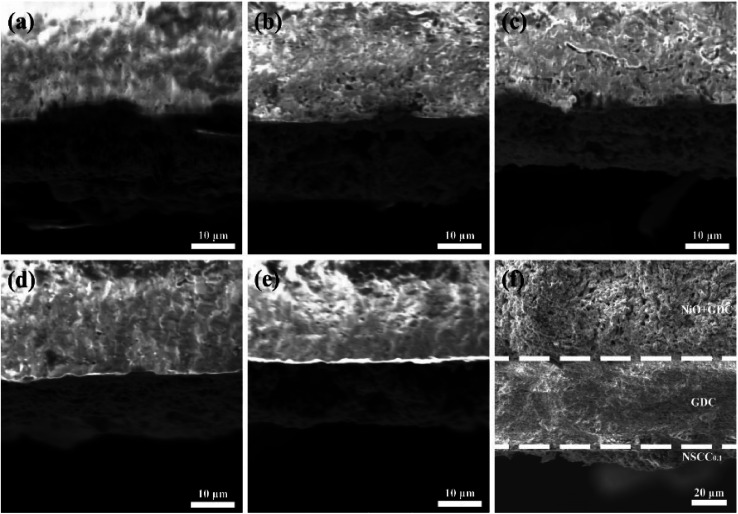
Cross section of symmetric battery with NSCC_*x*_ as cathode (a) NSCC, (b) NSCC_0.05_, (c) NSCC_0.1_, (d) NSCC_0.15_, (e) NSCC_0.2_, (f) single cell NSCC_0.1_|GDC|NiO-GDC.

The specific surface area and three-phase interface have direct influences on the cathode reaction.^21^ The SEM cross-section images of NSCC_*x*_|GDC are shown in Fig. 3(a)–(e) and NSCC_0.1_|GDC|NiO-GDC(f) are shown in Fig. 3(f). The upper side of Fig. 3(a)–(e) shows the microstructure of GDC electrolyte sintered at 1450 °C for 10 h, and the lower side is the microstructure of NSCC_*x*_ cathode. The microstructure of the samples with different Cu contents is highly similar, indicating that the introduction of Cu does not affect the electrochemical properties of the material by changing its microstructure.

**Fig. 4:**
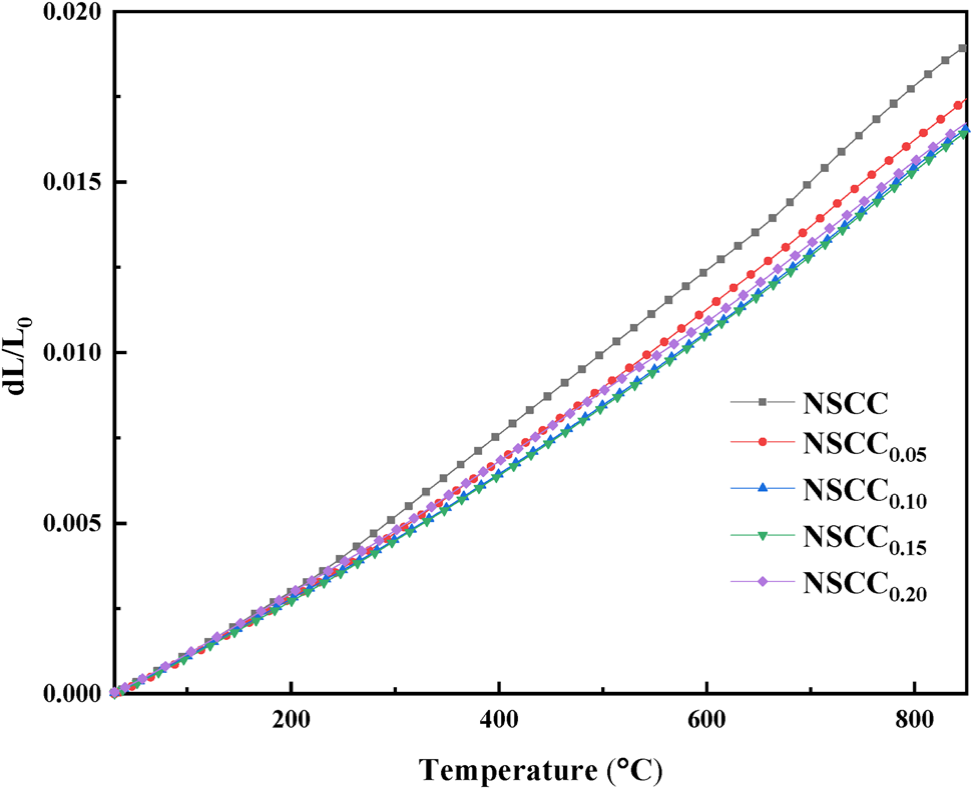
Temperature-dependence curves of TEC for NSCC_*x*_.

The variation of TEC with temperature for NSCC_*x*_ is shown in Fig. 4. With the increase in Cu content, the thermal expansion of the material is gradually inhibited. The average thermal expansion of NSCC_0.1_ is 20.2650 × 10^−6^ K^−1^, which is significantly lower than that of NSCC at 24.2049 × 10^−6^ K^−1^. On the one hand, the addition of Cu reduces the Co content in the material and consequently inhibits the thermal reduction of Co and restricts lattice expansion.^17^ On the other hand, the transformation of Co^3+^ from low spin Co^3+^ (t^6^_2g_e^0^_g_) to high spin Co^3+^ (t^4^_2g_e^2^_g_) and intermediate spin Co^3+^ (t^5^_2g_e^1^_g_) during heating accounts for the high TEC of cobalt-based perovskite oxides. Cu addition reduces the Co content and limits this transformation. Thus, the increase in TEC is suppressed.^22,23^ The main function of Cu doping is to reduce TEC and improve the catalytic effect on oxygen. Due to the high TEC of Co based materials, it is easy to cause electrode shedding in the high temperature environment after assembling full fuel cell. The reduction of TEC for Co based materials is aimed at matching the TEC of GDC electrolyte.^24^ Although it has little effect on the electrochemical performance, it will extend the application of Co based materials for commercial reasons.

**Fig. 5:**
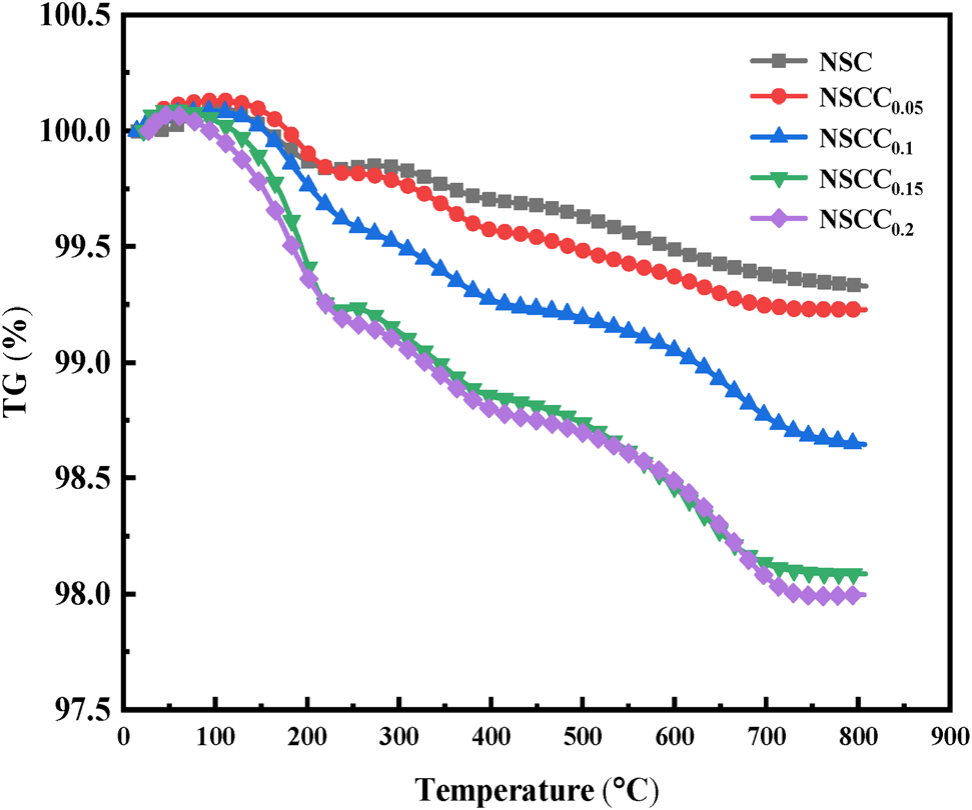
Temperature-dependence curves of NSCC_*x*_.

The thermogravimetric analysis results of NSCC_*x*_ in air are shown in Fig. 5. The samples show slow mass loss below 200 °C, which can be attributed to the decomposition and escape of water and CO_2_.^25^ At 250 °C–300 °C, a peak value appears in each curve, which can be considered as the mass increase caused by oxygen adsorption in the air.^26^ With the continuous increase in temperature, the high valence Co is reduced, and the Co–O bond fracture leads to the escape of lattice oxygen and the increase in oxygen vacancy. With the increase in Cu content, the Co–O bond length increases and the bond energy decreases. This bond easily breaks with the increasing temperature, resulting in the formation of additional oxygen vacancies.^27^ Furthermore, low valence Cu ion doping can produce additional oxygen vacancies, which is conducive to the improvement of cathode performance.

**Fig. 6:**
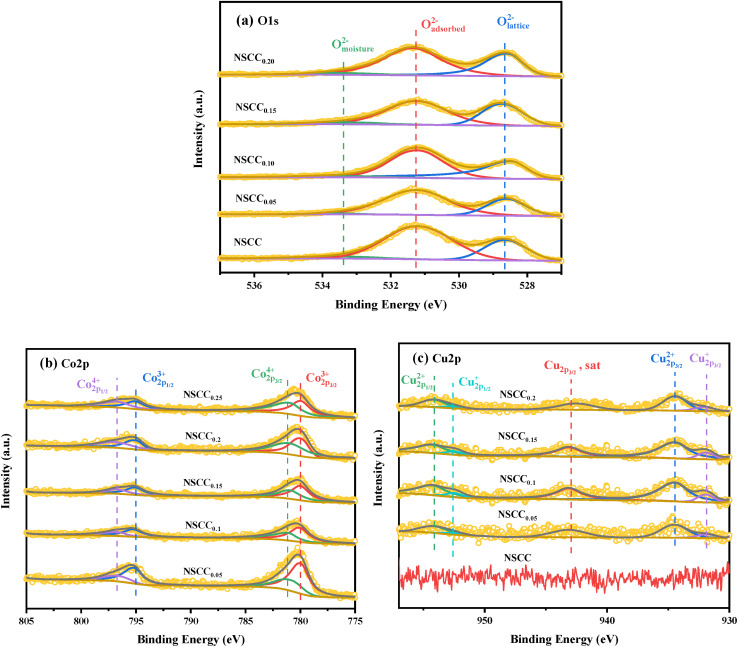
Fitted XPS spectra of NSCC_*x*_ (*x* = 0.05, 0.1, 0.15, 0.2): (a) O 1s, (b) Co 2p, (c) Cu 2p.

XPS was carried out on the samples to explore their surface ion valence state. The O 1s XPS curve and fitting results of NSCC_*x*_ samples are shown in Fig. 6(a). O 1s presents a high peak and a low peak and is divided into O_moisture_, O_adsorbed_, and O_lattice_.^9,28^ The ratio of O_adsorbed_/O_lattice_ was calculated to evaluate the oxygen vacancy generation capacity of each material, and the binding energy positions of peak are listed in Table 2. Obviously, O_adsorbed_/O_lattice_ ratio increases with the increase of Cu content, indicating that Cu addition can improve the oxygen vacancy content of the material. Oxygen vacancy is the main migration mode of oxygen ions in ORR. Owing to their high content of adsorbed oxygen content, Cu-containing materials exhibit improved oxygen migration and diffusion rate and electrochemical performance. Fig. 6(b) shows the XPS data and peak fitting results of Co 2p of each sample. The Co 2p_3/2_ peak is classified as Co^3+^ and Co^4+^ according to the binding energy of 779.9 ± 0.2 eV and 780.8 ± 0.2 eV, respectively. Co 2p_1/2_ peak is classified as Co^3+^ and Co^4+^ according to the binding energy of 794.9 ± 0.2 eV and 796.4 ± 0.2 eV, respectively. The binding energy locations and Co^3+^/Co^4+^ contents are shown in Table 3. According to the ratio of the two components, the increase in Cu content leads to the increase in Co^3+^ content and the decrease in Co^4+^ content, causing the improvement of electrochemical performance. Fig. 6(c) shows the XPS data and fitting results of Cu 2p. According to the binding energy, the peaks at 932.1 ± 0.2 eV and 952.4 ± 0.2 eV belong to Cu^+^, and those at 934.3 ± 0.2 eV and 954.3 ± 0.2 eV belong to Cu^2+^. Therefore, Cu exists in the form of Cu^+^ and Cu^2+^ in the NSCC_*x*_ samples.

**Table 2:** The binding energy of O_moisture_, O_adsorbed_, O_lattice_ and the ratio of O_adsorbed_/O_lattice_

Sample	O_moisture_ (eV)	O_adsorbed_ (eV)	O_lattice_ (eV)	O_adsorbed_/O_lattice_
NSCC	533.49	531.18	528.64	1.50
NSCC_0.05_	533.50	531.20	528.61	1.70
NSCC_0.1_	533.49	531.20	528.49	1.76
NSCC_0.15_	532.50	531.24	528.69	2.47
NSCC_0.2_	532.51	531.34	528.58	2.58

**Table 3:** The Co 2p binding energy and content of Co^4+^ and Co^3+^ calculated from the corresponding XPS peaks

Sample	Co_2p_1/2__^4+^ (eV)	Co_2p_1/2__^3+^ (eV)	Co_2p_3/2__^4+^ (eV)	Co_2p_3/2__^4+^ (eV)	Co^4+^ (%)	Co^3+^ (%)
NSCC	796.53	795.05	780.87	779.95	73.77	26.23
NSCC_0.05_	796.38	794.82	780.64	779.75	65.32	34.68
NSCC_0.1_	796.30	794.89	780.66	779.84	62.83	37.17
NSCC_0.15_	796.30	794.89	780.65	779.89	61.80	38.20
NSCC_0.2_	796.64	795.02	780.78	779.95	57.12	42.88

**Fig. 7:**
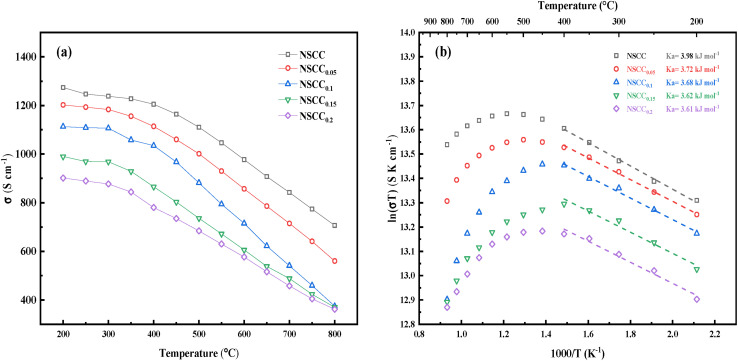
(a) Temperature dependence of conductivity for NSCC_*x*_ (b) Arrhenius curve of NSCC_*x*_.

The conductivity of NSCC_*x*_ in the temperature range of 200 °C–800 °C was tested by DC four-electrode method, as shown in Fig. 7(a). The conductivity of the material decreases slowly before 400 °C, implying its semiconductor characteristics, but decreases rapidly in the range of 400 °C–800 °C, implying its metal conduction behavior. In this temperature range, the conductivity of the material decreases with the increase in Cu content. Cu substitution for Co inevitably decreases the number of holes, thus reducing the Co^4+^/Co^3+^–O–Co^3+^/Co^2+^ hopping path of the small polaron and weakening the proton transfer rate of the sample.^29,30^ Meanwhile, Cu introduction has a slight strengthening effect on oxygen ion conduction. Under the interaction of the two mechanisms, Cu introduction decreases the conductivity. The conductance Arrhenius diagram in Fig. 7(b) presents a straight line in the low temperature range, indicating that each sample follows the small polaron hopping conductive mechanism, and electrons are transmitted along the Co^4+^/Co^3+^–O–Co^3+^/Co^2+^ path.^31,32^ The apparent activation energy *E*_a_ of the conductivity was calculated according to Arrhenius formula (eqn (1)) to further quantify the difficulty of small polaron transmission, and the result is shown in Fig. 7(b).1
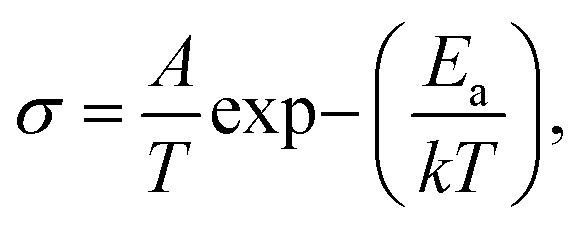
where *A* is the pre-factor, *T* is the absolute temperature, *K* is the Boltzmann constant, and *E*_a_ is the activation energy. With the increase in Cu content, the *E*_a_ of the sample decreases from 3.98 kJ mol^−1^ for NSCC to 3.68 kJ mol^−1^ for NSCC_0.1_. This phenomenon occurs because the increase in Cu also increases the oxygen vacancy concentration, thus reducing the apparent activation energy of the conductance and increasing the conduction and diffusion rate of oxygen ions.^33^ With further increase in Cu content, the decline in *E*_a_ decreases slowly to 3.61 kJ mol^−1^ for NSCC_0.2_. Therefore, the new unknown phase formed by excessive Cu provides no improvement to oxygen ions and even acts as an obstacle in the small polar ion hopping path.

**Fig. 8:**
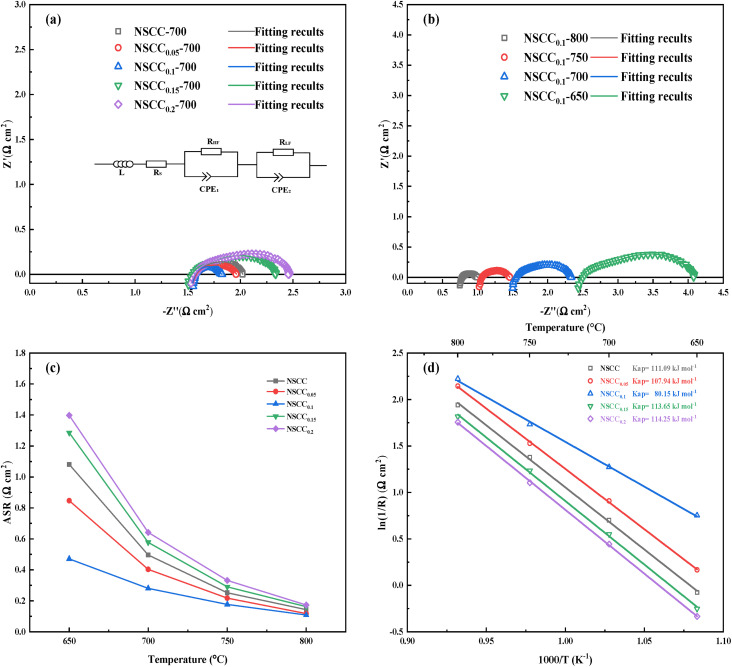
(a) Nyquist diagram of symmetric battery with NSCC_*x*_ as cathode at 700 °C; (b) Nyquist diagram of NSCC_0.1_ at 650–800 °C; (c) relationship between ASR and temperature; (d) polarization arrhenius diagram of symmetric cell with NSCC_*x*_ as cathode.

EIS was measured in constructed symmetrical cells. Fig. 8(a) is the Nyquist diagram of NSCC_*x*_ with different Cu contents at 700 °C. The curves of each sample are semi-arcs with different radii, and the kinetic steps are usually divided according to the frequency range. The high frequency band corresponds to the charge transfer of oxygen ions at the interface between the electrode and electrolyte, and the low frequency band corresponds to the adsorption and dissociation of oxygen. With the increase in Cu content, the curve radius of each sample decreases first and then increases, indicating that the impedance value of the material decreases first and then rises. Fig. 8(b) is the Nyquist diagram of NSCC_0.1_ at different temperatures. The impedance value decreases with the increase in temperature, and the ORR activity increases significantly at high temperature. These findings indicated that temperature is the main factor affecting the oxygen catalytic activity of NSCC_*x*_. The EIS data of the samples were further analyzed by equivalent circuit method as shown in Fig. 8(a). The area specific resistance (ASR) of each material was obtained after calculation and normalization based on the fitting data as shown in Fig. 8(c). With the increase in Cu content, the ASR decreases first and then increases. NSCC_0.1_ always has the lowest ASR, indicating its optimal ORR activity among the NSCC_*x*_ samples. Meanwhile, according to the equivalent circuit, the contribution of its ASR value comes from the equivalent resistors RHF and RLF in the two frequency ranges (Table 4). It can be seen that the decrease of resistance mainly occurs in the low frequency band, indicating that the addition of Cu strengthens the mass transfer process of the cathode material, but has a limited effect on the charge transfer process. At 800 °C, the ASR value of NSCC_0.1_ is 0.108 Ω cm^2^, showing a lower ASR than La_0.6_Sr_0.4_Co_0.2_Fe_0.8_O_3−*δ*_ (0.18 Ω cm^2^) and better electrochemical performance.^34^

**Table 4:** Polarization resistance results of NSCC_*x*_ materials as the symmetric electrode at 700 °C in air

Sample	ASR(Ω cm^2^)	*R*_HF_ (Ω cm^2^)	*R*_LF_ (Ω cm^2^)
NSCC	0.50	0.22	0.27
NSCC_0.05_	0.40	0.20	0.20
NSCC_0.1_	0.28	0.17	0.11
NSCC_0.15_	0.58	0.23	0.35
NSCC_0.2_	0.64	0.25	0.39

Furthermore, the Arrhenius diagram of symmetric fuel cell with NSCC_*x*_ as the cathode was drawn based on ASR, and *E*_a_ was calculated as shown in Fig. 8(d). NSCC_0.1_ has the lowest *E*_a_ of 80.15 kJ mol^−1^ because Cu addition increases the amount of oxygen vacancies, provides additional paths for oxygen ion transport, and improves the adsorption/dissociation capacity of oxygen ions. The results showed that the ORR activity of the material was increased by the addition of Cu. With further increase in Cu content, the *E*_a_ decreases because the newly formed phase hinders the oxygen ion transport and destroys the original oxygen transport structure.

**Fig. 9:**
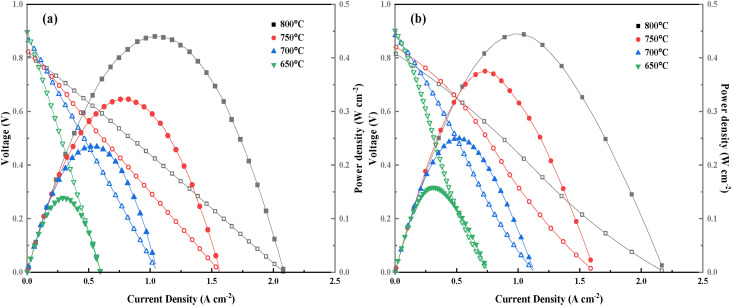
The curves of output (a) NSCC; (b) NSCC_0.1_.

The output power of a single cell was tested to investigate the change of the electrochemical performance induced by Cu doping. Fig. 9 shows the output power curves of NSCC(a) and NSCC_0.1_(b). With the increase in operating temperature, the open circuit voltage decreases from 0.9 V to about 0.8 V. At 800 °C, the peak output power of NSCC_0.1_ is 444.87 mW cm^−2^, which is slightly better than that NSCC at 440.10 mW cm^−2^ as shown in Table 5. These results showed that Cu doping can improve the stability of the material without weakening its properties. Hence, NSCC_0.1_ can be used as a SOFC cathode.

**Table 5:** Power density of NSCC and NSCC_0.1_ in range of 650–800 °C

Sample	800 °C (mW cm^−2^)	750 °C (mW cm^−2^)	700 °C (mW cm^−2^)	650 °C (mW cm^−2^)
NSCC	440.10	323.51	234.94	139.24
NSCC0.1	444.87	375.26	249.76	158.39

## 4. Conclusion

Cu-doped Nd_0.6_Sr_0.4_Co_1−*x*_Cu_*x*_O_3−*δ*_ (*x* = 0, 0.05, 0.1, 0.15, 0.2) perovskite oxide material was successfully synthesized. The experimental results showed that with the increase in Cu doping content, the lattice expands gradually and becomes a single orthonormal structure when *x* ≤ 0.1. The average TEC of NSCC_0.1_ decreases by 16.28% when Cu replaces Co at 35 °C–800 °C. The conductivity of NSCC_*x*_ decreases gradually with the increase in Cu content, and the conductivity of NSCC_0.1_ is 541 S cm^−1^ at 800 °C. EIS showed that the ASR at 700 °C is 0.1619 Ω cm^2^, which is 11.04% lower than that for NSCC. At 800 °C, the peak output power density of NSCC_0.1_ is 444.87 mW cm^−2^. Therefore, NSCC_*x*_ can be used as a candidate material for intermediate-temperature SOFC cathode.

## Conflicts of interest

There are no conflicts to declare.

## Supplementary Material
